# Construction and validation of a predictive model for lymph node metastasis in patients with papillary thyroid carcinoma

**DOI:** 10.3389/fendo.2025.1551108

**Published:** 2025-06-09

**Authors:** Yanhong Hao, Yanjing Zhang, Yuan Su, Liping Liu

**Affiliations:** ^1^ Department of Ultrasound, The First Hospital of Shanxi Medical University, Taiyuan, China; ^2^ Department of Interventional Ultrasound, The First Hospital of Shanxi Medical University, Taiyuan, China; ^3^ Department of Imaging Medicine, Shanxi Medical University, Taiyuan, China

**Keywords:** papillary thyroid cancer, lymphonodi cervicales metastasis, machine learning, prediction model, SHAP

## Abstract

**Objective:**

To study the occurrence of lymph node metastasis in patients with papillary thyroid carcinoma (PTC) and construct a predictive model to assess its predictive performance.

**Methods:**

We retrospectively analyzed the data of 432 patients with PTC. The least absolute shrinkage and selection operator (LASSO) was used to select the features, and multiple logistic regression was used to analyze the predictive factors. Multiple machine learning (ML) classification models are integrated to analyze and identify the optimal model, while Shapley additive exPlanations (SHAPs) are used for personalized risk assessment. A total of 125 patients from Changzhi Heping Hospital were included in an external validation set to evaluate the generalizability of our model.

**Results:**

Predictors of central lymph node metastasis (CLNM) included age, sex, maximum nodule diameter, margin, morphology, number of nodules, relationship between the nodule and the thyroid envelope, and coarse calcification. A logistic classification model was identified as the optimal model, with a test set area under the curve (AUC) value of 0.798. The validation results using external data were consistent, demonstrating the stability and generalizability of our model.

**Conclusion:**

We established a logistic model using the SHAP method, which provides evidence for the ability of the SHAP method to predict lymph node metastasis and serves as a basis for personalized healthcare.

## 1 Background

Papillary thyroid carcinoma (PTC) is the most common histological subtype of thyroid cancer and its incidence among women in Asian countries has increased significantly since 2000 (1). PTC typically follows an indolent clinical course with a favorable prognosis; however, patients with lymph nodule metastasis have an elevated risk of local recurrence. As the N stage increases, the likelihood of distant metastasis significantly increases, leading to a poorer prognosis (2). Studies have confirmed that total thyroidectomy provides a low local recurrence benefit; therefore, preventive central lymph node dissection is not necessary unless lymph nodule metastasis is detected before surgery (3). Ultrasound is an easy and cost-effective method for evaluating lymph node metastasis in PTC, but its sensitivity for detecting central lymph node involvement is low due to anatomical limitations and the frequent absence of abnormalities on preoperative imaging (4). Therefore, early identification of risk factors and construction of prediction models are highly important for improving the early prediction of lymph node metastasis.

Machine learning (ML) is a new and powerful tool in the field of medicine, particularly in personalized medicine and computer-aided diagnosis (5). In this study, various ML classification models were used to create predictive models. We gathered and analyzed clinical data from patients with PTC to understand the factors influencing lymph node metastasis and guide surgical treatment. In addition, interpretation of the model is challenging. For more intuitive results, the Shapley additive exPlanations (SHAP) tool was used to visually interpret the risk factors influencing patient predictions (5). SHAP not only quantifies individual probabilities of clinical events, but also integrates biological and clinical models, thereby contributing to the advancements of personalized medicine. Therefore, we aimed to develop a more accurate prediction model for lymph nodule metastasis in patients with PTC based on clinical data.

## 2 Materials and methods

We conducted a retrospective study of 432 patients with surgically and pathologically confirmed papillary thyroid carcinoma (PTC) treated at our hospital between January 2020 and October 2021. Concurrently, 125 patients from Changzhi Heping Hospital were used as an external validation set. Based on the pathological findings, all patients were divided into positive and negative lymph node metastasis groups. The inclusion criteria were as follows: 1) surgically confirmed PTC with cervical lymph node pathology records, 2) thyroid ultrasonography recorded within 2 weeks before surgery, and 3) laboratory examination of thyroid serum markers within 2 weeks before surgery. The exclusion criteria were as follows: 1) incomplete clinical data, and 2) unclear ultrasound images or incomplete data for analysis. The study was conducted in accordance with the principles of the Declaration of Helsinki.

### 2.1 Methods

#### 2.1.1 US equipment and US characteristics

Ultrasonographic examination was performed via Canon i800 color Doppler ultrasound equipment, and the robe frequency was 18 MHz. The ultrasonographic features included the location, maximum diameter, echogenicity, margin, morphology, relationship of the nodule to the thyroid capsule (distant from the thyroid capsule (≥2 mm), in contact with the thyroid capsule (<2 mm), and invasion or penetration of the thyroid capsule), aspect ratio, nature of the nodule, intranodal calcifications (≤2 mm is defined as microcalcification, and >2 mm is defined as macrocalcification) and the number of nodules. Irregular margins included irregular, ill-defined, nodular, and lobular features. All ultrasound images were assessed by a sonographer who had been performing ultrasound examinations for more than five years, and the ultrasound features were analyzed by a senior doctor.

#### 2.1.2 Clinical and serological indicators of thyroid function

Age, sex, and thyroid serological parameters, including triiodothyronine (FT3), serum-free thyroxine (FT4), thyroid-stimulating hormone (TSH), thyroglobulin (Tg), antithyroglobulin antibody (TG-Ab), and anti-thyroid peroxidase antibody (TPOAb), were collected from the patients during the first 2 weeks of the operation. The TPOAb level was considered normal if it was within the normal range and high if it was above normal. Hashimoto’s thyroiditis (TH) was defined based on the positive level of the patient’s own TPOAb before the operation and changes in the characteristics of the ultrasound image.

#### 2.1.3 BRAF^V600E^ gene mutation detection

Thyroid nodules with the most apparent signs of malignancy, as assessed by conventional ultrasound, were selected for ultrasound-guided fine needle aspiration, with four punctures per lesion to ensure an adequate tissue sample volume. Genetic and cytological samples were placed in specimen tubes and liquid-based vials, respectively, and DNA was extracted using commercial kits. BRAF^V600E^ mutation was detected using a real-time fluorescence quantitative polymerase chain reaction amplification-based kit.

#### 2.1.4 Surgical management in study protocol

All enrolled patients underwent standardized thyroidectomy with nodal dissection guided by preoperative ultrasound risk stratification: unilateral lobectomy for localized tumors (T1-2) versus total thyroidectomy for multifocal/aggressive variants (6), complemented by prophylactic cervical lymph node dissection and therapeutic lateral neck dissection for ultrasound-suspected nodes. Therapeutic lateral neck dissection encompassing levels II-V was systematically performed when intraoperative frozen sections confirmed metastasis in ultrasonography-suspected lymph nodes, adhering to the compartment-oriented dissection principles outlined in the 2015 ATA guidelines.

#### 2.1.5 Establishment and evaluation of predictive models

Patients were randomly divided into training and testing groups in an 8:2 ratio, and characteristic factors were selected within the training set. Least absolute shrinkage and selection operator (LASSO) is employed for variable selection, which help mitigate overfitting by shrinking the variable coefficients, and effectively addresses issues of severe multicollinearity. Multiple machine learning (ML) classification models were used for a comprehensive analysis, the importance of each indicator in the training group was compared, and the testing group was evaluated using different models. In addition, we evaluate and validate the results using an optimal model. Shapley additive exPlanations (SHAP) visualizes the overall presentation model and individual sample interpretations. The specific steps were as follows. (1) Data division: Using the random number method in SPSS, patients with PTCs were randomly divided into a training set and a test set at a ratio of 8:2, with 348 cases in the training set and 84 cases in the test set. (2) Screening of characteristic factors: First, LASSO regression analysis was performed using R software (glmnet4.1.8) for variable selection and complexity adjustment. Then based on the results of the LASSO regression analysis, multifactor logistic regression analysis was conducted using SPSS to identify characteristic factors with statistical significance (*p*<0.05). (3) Python was used to perform a comprehensive analysis of multiple classification models, including logistic regression, light gradient boosting machine (LightGBM), random forest, adaptive boosting (AdBoost), decision tree classification, gradient boosting decision tree (GBDT) classification, multilayer perceptron (MLP), support vector machine (SVM), and Gaussian naive Bayes (GNB) methods. Repeated sampling validation was performed using R, with a validation set ratio of 0.3, 10 validation iterations, and a random seed of 42 for model training and validation. The aforementioned parameterized models (with 10 repetitions of sampling) were trained and tested. The importance of indicators in the training and test sets was analyzed across different models, and the optimal model was selected. Python (sklearn 0.22.1) was used to construct the area under the receiver operating characteristic (ROC) curve, and R software (rmda 1.6) was used to perform decision curve analysis (DCA). Python (sklearn 0.22.1) was employed to generate the calibration curve for assessing the predictive ability of the model, and to conduct a comprehensive evaluation of the predictive model to verify its usefulness in decision support and broader simulation modeling. Python (sklearn 0.22.1) was used to plot the precision–recall (PR) curve, which is widely used to evaluate model performance. (4) Training, validation, and testing of the optimal model: Performed 10-fold cross-validation on the training set and evaluated with the test set. Python (sklearn 0.22.1) was used to plot learning curves to assess the model fit and stability of the training and validation sets. (5) Python (shape 0.39.0) was used for the SHAP-based interpretation to analyze model importance and feature contributions. The contribution of each feature to the prediction results was calculated to explain the model’s outputs. Additionally, the SHAP values were generated for individual samples, and predictive performance was evaluated. Data from Heping Hospital were employed to externally validate the optimal model. Linear predictors (LPs) were first calculated for the external validation dataset. This model was then evaluated against the external dataset, and both ROC and calibration curves were generated to assess the generalization capability of our model.

### 2.2 Statistical analysis

SPSS 23.0, Python (version 3.4.3), R (version 3.6.1), and Free Statistics version 2.1 (http://www.clinicalscientists.cn/freestatistics/, Beijing, China) were used to analyze the data. All clinical data and ultrasound features in the training and test groups were compared at baseline. Because continuous variables did not follow a normal distribution, they were represented as median [IQR], while categorical variables were represented as n(%). Comparisons between groups were performed using the Mann-Whitney U test, and categorical variables were compared using the chi-square test. *p*<0.05 was considered statistically significant.

## 3 Results

### 3.1 Comparison of baseline data

A total of 432 patients were randomly divided into training and testing groups of 348 and 84 patients, respectively, using the random number method (8:2 test ratio). There was no significant difference in the baseline data between the two groups (*p*>0.05) (**Table 1**). In the subgroups of CLNM and LCLNM, only the size of the nodules demonstrated a significant difference (as shown in **Supplementary Table S1**).

**Table 1:** Comparison of baseline characteristics between the two groups.

Variable	N = 432^1^	Testing group N = 84	Training group N = 348	*p*
Age (year)	46.000 (36.000 – 54.000)	45.500 (37.000 – 53.000)	46.000 (36.000 – 55.000)	0.814
Gender				0.423
Male	94 (22)	21 (25)	73 (21)	
Female	338 (78)	63 (75)	275 (79)	
Size (cm)	0.785 (0.630 – 1.275)	0.790 (0.660 – 1.305)	0.775 (0.620 – 1.263)	0.567
BRAF^V600E^ mutation				0.456
No	40 (9.3)	6 (7.1)	34 (9.8)	
Yes	392 (91)	78 (93)	314 (90)	
FT3				>0.999
Normal	408 (94)	80 (95)	328 (94)	
Abnormal	24 (5.6)	4 (4.8)	20 (5.7)	
FT4				0.861
Normal	403 (93)	78 (93)	325 (93)	
Abnormal	29 (6.7)	6 (7.1)	23 (6.6)	
TSH				0.515
Normal	333 (77)	67 (80)	266 (76)	
Abnormal	99 (23)	17 (20)	82 (24)	
Tg				0.227
Normal	396 (92)	75 (89)	321 (92)	
Mild abnormality	19 (4.4)	3 (3.6)	16 (4.6)	
Significant abnormality	17 (3.9)	6 (7.1)	11 (3.2)	
HT				0.678
No	380 (88)	75 (89)	305 (88)	
Yes	52 (12)	9 (11)	43 (12)	
Margin				0.213
Clear	50 (12)	13 (15)	37 (11)	
Unclear	382 (88)	71 (85)	311 (89)	
Relationship to capsule				0.377
Away from	137 (32)	24 (29)	113 (32)	
Adjacent	182 (42)	33 (39)	149 (43)	
Invasion	113 (26)	27 (32)	86 (25)	
Lymphonodi cervicales metastasis				0.489
No	235 (54)	46 (55)	189 (54)	
Yes	197 (45)	38 (46)	159 (45)	
Number				0.913
Solitary	244 (56)	47 (56)	197 (57)	
Multiple	188 (44)	37 (44)	151 (43)	
Composition				0.885
Solid	394 (91)	78 (93)	316 (91)	
Mixed solid and cystic	38 (8.8)	6 (7.2)	23 (9.2)	
Echogenicity				0.304
Hypo	388 (90)	78 (93)	310 (89)	
Isoechoic or hyper	44 (10)	6 (7.1)	38 (11)	
Coarse calcification				0.087
No	301 (70)	65 (77)	236 (68)	
Yes	131 (30)	19 (23)	112 (32)	
Aspect ratio				0.461
<1	100 (23)	22 (26)	78 (22)	
≥1	332 (77)	62 (74)	270 (78)	
Shape				0.688
Regular	126 (29)	26 (31)	100 (29)	
Irregular	306 (71)	58 (69)	248 (71)	
Microcalcification				0.207
No	77 (18)	11 (13)	66 (19)	
Yes	355 (82)	73 (87)	282 (81)	
Position (upper-lower distribution)				0.162
Isthmus	18 (4.2)	6 (7.1)	12 (3.4)	
Upper	69 (16)	8 (9.5)	61 (18)	
Middle	183 (42)	38 (45)	145 (42)	
Lower	82 (19)	19 (23)	63 (18)	
Diffuse occurrence	80 (19)	13 (15)	67 (19)	
Location (left-right distribution)				0.406
Isthmus	18 (4.2)	6 (7.1)	12 (3.4)	
Left	141 (33)	26 (31)	115 (33)	
Right	193 (45)	39 (46)	154 (44)	
Diffuse occurrence	80 (19)	13 (15)	67 (19)	

Data are presented as median (interquartile range) for continuous variables and number (percentage) for categorical variables. *p*-values were calculated using the Mann-Whitney U test for continuous variables and the Chi-square test for categorical variables.

### 3.2 Risk factor screening for lymph node metastasis in patients with papillary thyroid carcinoma

**Figure 1:**
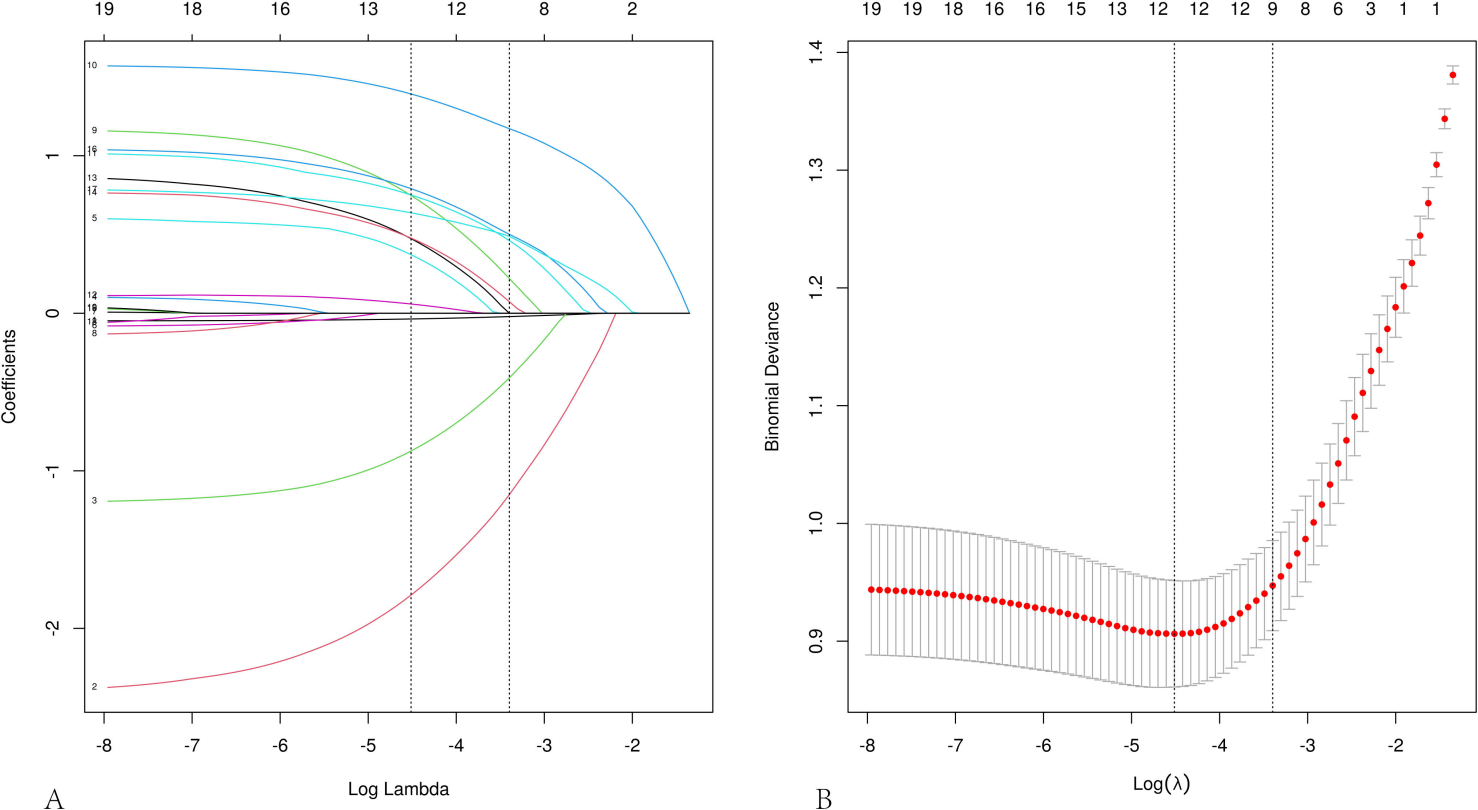
Feature selection using LASSO regression analysis. **(A)** Coefficient profile plot. **(B)** LASSO regression cross-validation curve, plotted in the LASSO model with the minimum mean squared error (lambda = 0.011) and the minimum standard error of the mean (lambda = 0.033).

LASSO regression analysis was performed on the aforementioned independent variables with cervical lymph node metastasis as the dependent variable to establish a LASSO regression model (**Figure 1**). The results show that the optimal λ value with the minimum mean squared error is 0.033, reducing the number of independent variables from 21 to 11, including nodule size, age, sex, FT4, margin, adjacent capsule, capsular invasion, number, coarse calcifications, microcalcifications, and nodule shape. To further control for the influence of confounding factors, a multivariate logistic regression analysis was conducted on the 11 independent variables. Only nodule size, age, sex, margins, adjacent capsules, capsular invasion, number, coarse calcifications, and nodule shape were identified as a significant risk factors (**Table 2**). Receiver operating characteristic (ROC) curves were used to assess the predictive value of age and nodal size for lymph node metastasis. The results showed that the area under the curve (AUC) for patient age and the maximum nodule diameter were 0.615 and 0.725, respectively. The optimal cutoff values were 45.0 years for age and 0.75 cm for the nodule diameter (**Table 3**).

**Table 2:** Multifactorial logistic regression for lymphovascular cervical metastasis.

Predictor	Estimate	SE	Z	*p*	Odds ratio	Lower	Upper
(Intercept)	-1.99	1.01	-1.96	0.05	0.14	0.02	0.95
Age	-0.06	0.01	-4.57	0.0	0.94	0.91	0.96
Gender	-1.13	0.37	-3.08	0.0	0.32	0.16	0.65
Size	0.81	0.27	2.93	0.0	2.24	1.35	3.96
FT4	0.67	0.57	1.17	0.24	1.95	0.64	6.2
Margin	1.29	0.65	1.98	0.05	3.65	1.08	14.52
Adjacent	2.01	0.39	5.16	0.0	7.49	3.58	16.65
Invasion	2.76	0.46	6.06	0.0	15.78	6.67	39.97
Number	0.62	0.3	2.08	0.04	1.86	1.04	3.36
Coarse calcification	0.82	0.32	2.57	0.01	2.28	1.22	4.31
Shape	0.95	0.35	2.67	0.01	2.58	1.3	5.25
Microcalcification	0.62	0.4	1.55	0.12	1.86	0.86	4.16

**Table 3:** Diagnostic performance of age and nodule diameter.

Characterization	AUC	Sensitivity	Specificity	Jordon’s index	Optimal thresholds
Age	0.615	0.641	0.58	0.221	45.0
Maximum diameter	0.725	0.745	0.605	0.35	0.75

### 3.3 Comprehensive analysis of the classified multi-model

**Figure 2:**
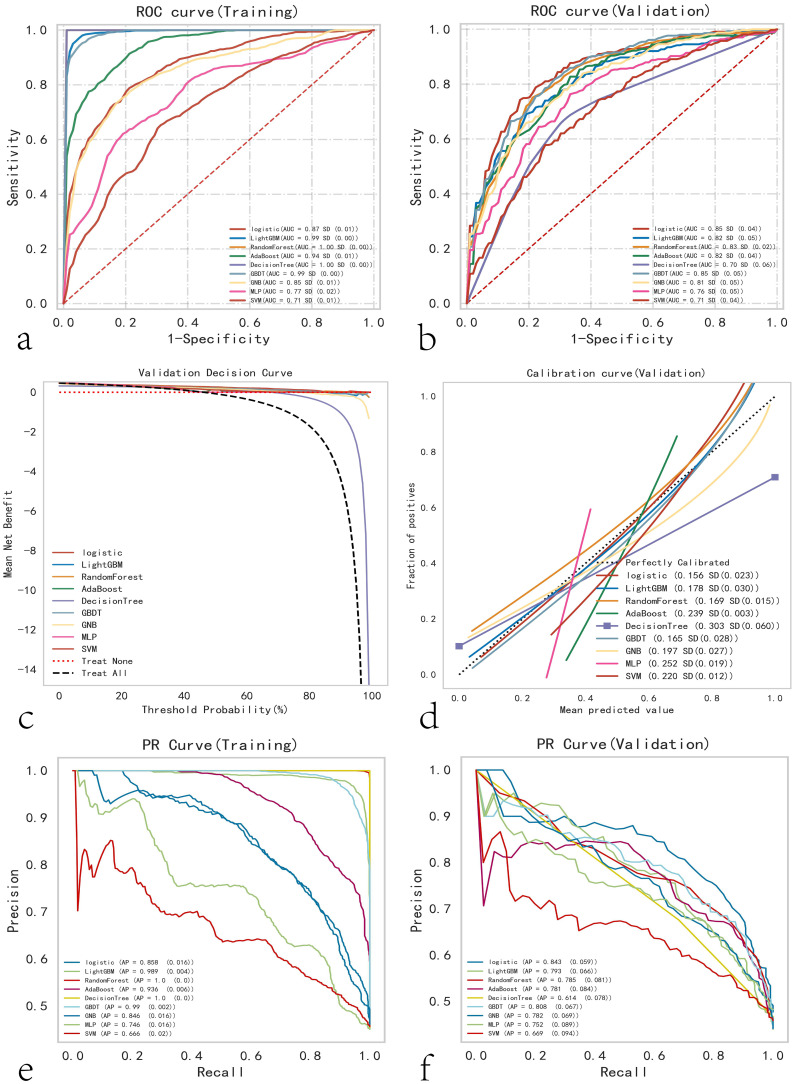
Comprehensive analysis of ML (Machine Learning) models. **(a)** Training set ROC and AUC, and **(b)** test set ROC and AUC. Papillary thyroid carcinoma patients were sampled 10 times in an 8:2 ratio. **(c)** Test set DCA, where the black dashed line represents the assumption that all patients have cervical lymph node metastasis, and the red dashed line and thin black line represent the assumption that no patients have cervical lymph node metastasis. The remaining solid lines represent different models. **(d)** Test set calibration curves, with the horizontal axis representing the average predicted probability and the vertical axis representing the actual probability of the event. The dashed diagonal line is the reference line, and other smooth solid lines are the fitting lines for different models. The closer the fitting line is to the reference line and the smaller the value in parentheses, the more accurate the model’s predicted values. **(e)** Training set PR curve and AP, and **(f)** test set PR curve and AP, where the y-axis is precision and the x-axis is recall. If the PR curve of one model is completely covered by the PR curve of another model, it can be concluded that the latter is superior to the former. The higher the AP value, the better the model performance. Different colors in the figure represent the corresponding models.

The predictive performance of various machine learning models was assessed using the AUC. The results indicated that in the training cohort, Random Forest, LightGBM, and GBDT performed the best, whereas in the testing cohort, Logistic Regression was optimal (**Figures 2a, b**). The clinical applicability of different models was further evaluated using DCA (**Figure 2c**), calibration curves (**Figure 2d**), and PR curves. Calibration curves demonstrated that the Logistic Regression model exhibited higher predictive accuracy. In the training set, the logistic model demonstrated superior performance; in the test set, it showed the best performance, with the highest AP value in the validation set (**Figures 2e, f**). A comprehensive analysis indicated that the logistic model could be considered optimal.

### 3.4 Construction and evaluation of the optimum model

**Figure 3:**
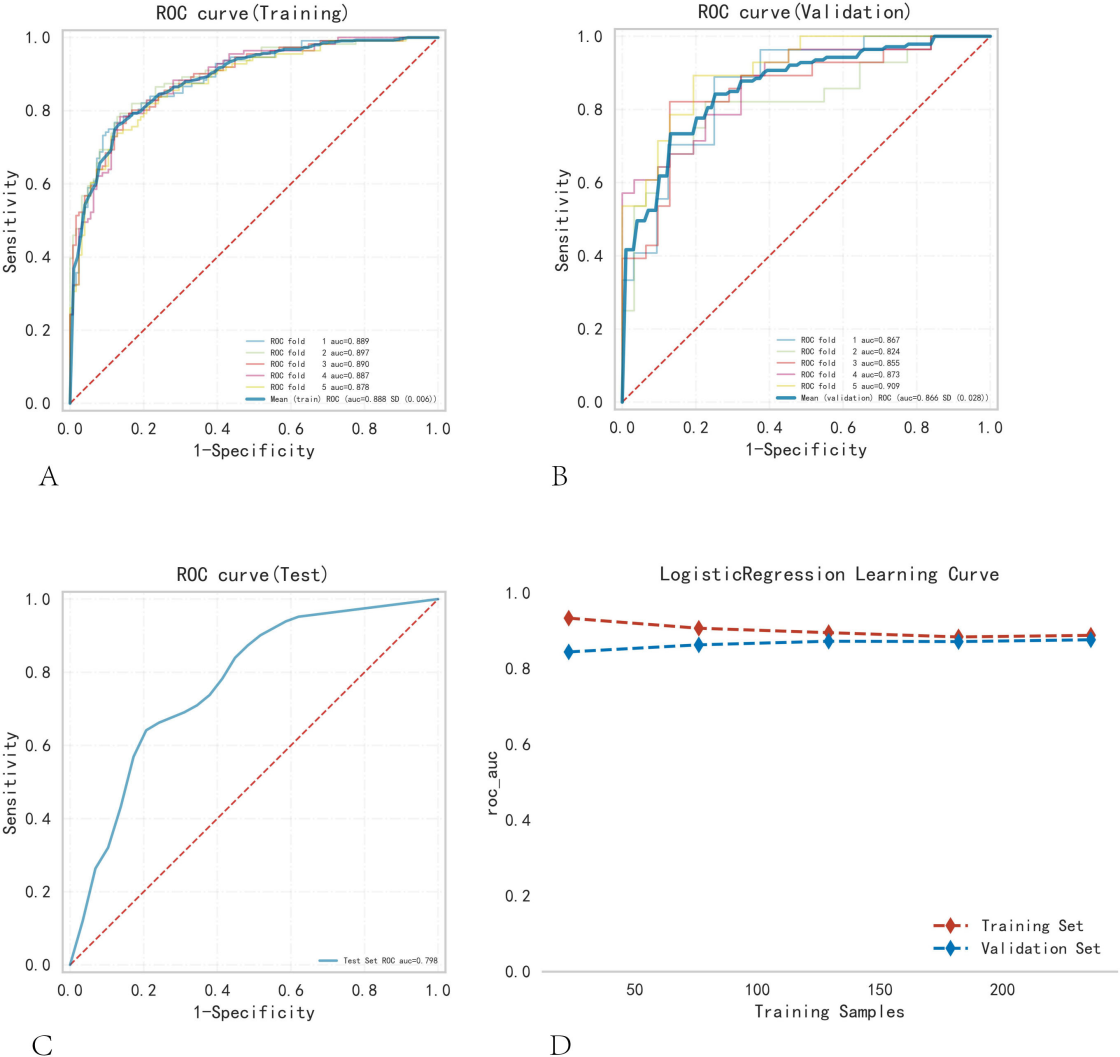
Training, validation, and testing of the logistic regression model. **(A)** Training set ROC and AUC, and **(B)** validation set ROC and AUC. Training and cross-validation were conducted on 10% of PTC patients, with different colored solid lines representing different outcomes. **(C)** Test set ROC and AUC. **(D)** Learning curve. The red dashed line represents the training set, and the blue dashed line represents the validation set.

Logistic regression analysis and a 10-fold cross-validation test were performed, and the results were verified using the training set. The results confirmed that the mean AUC of the validation and testing groups were 0.888, 0.866, and 0.798 (**Figures 3A–C**). The AUCs of the three groups eventually stabilized at approximately 0.85, and the model predictions were accurate. Because the performance of the validation set was lower than that of the test set in terms of the AUC metric or ratio being less than 10%, the model fitting was considered successful. The learning curve indicated that the training and validation sets had a strong fit and high stability (**Figure 3D**). These outcomes suggest that the logistic regression model can be used for classification modeling tasks in the dataset. In different subgroups, the model exhibits excellent discrimination, with AUC values of 0.879 for CLNM and 0.781 for LCLNM (as shown in **Supplementary Table S2**; **Figure S1**). The model achieved an AUC of 0.876 in the external validation dataset, confirming its robustness and generalizability (**Supplementary Figure S1**).

### 3.5 SHAP to model visual explanations

**Figure 4:**
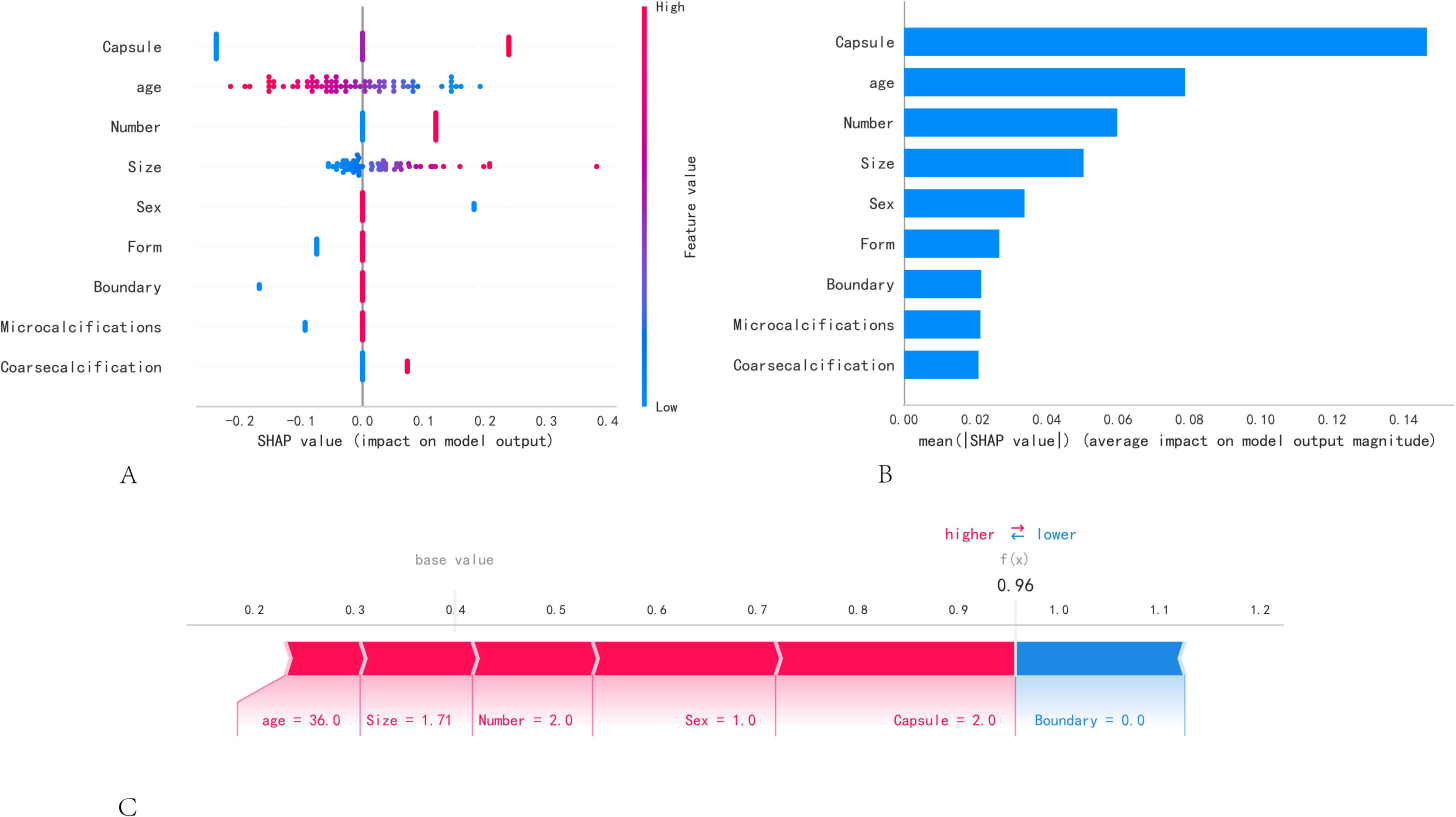
SHAP model interpretation. **(A)** Feature attributes in SHAP. Each row represents an element, and the horizontal axis is the SHAP value. Red dots indicate higher feature values, and blue dots indicate lower feature values. **(B)** Feature importance ranking as shown by SHAP. The matrix plot describes the importance of each covariate in the development of the final predictive model. **(C)** A specific case demonstration.

**Figure 4A** employs SHAP to interpret the role of the nine variables in our model in predicting the status of lymph node metastasis in PTC. Different colored dots represent the attribution of different features to the outcome; red dots represent high-risk values, and blue dots represent low-risk values. The occurrence of CLNM increased with capsule invasion, age < 45 years, the presence of multiple nodes, nodule size, and male sex. **Figure 4B** shows the ranking of the nine risk factors assessed using the average absolute SHAP values, with the SHAP values on the x-axis indicating the importance of the predictive model. Moreover, we provided a typical CLNM+ case to illustrate the interpretability of the model. The SHAP predictive score was 0.96. The positive act of each feature is shown; red stripes indicate positive actions, and blue stripes indicate inactive actions (**Figure 4C**).

## 4 Discussion

The incidence rate of PTC has increased in recent years, particularly for tumors with a maximum diameter of <1 cm (7). Traditional surgery involves prophylactic central compartment lymph node dissection; however, unnecessary central compartment lymph node dissection can lead to recurrent laryngeal nerve injury. Moreover, the surgical scope for the lateral cervical lymph nodes is larger, which can result in more complications and reduce the quality of life of patients after surgery (8). In this study, the rate of cervical lymph node diversion was as high as 46%. Therefore, effective preoperative prediction of lymph node metastasis can help guide the development of clinical surgical protocols.

Research on ML has always been a key focus in the medical field. We employed several ML models and found that the logistic model generally outperforms the others, based on analyses of the AUC, DCA, PR curves, and calibration curves. However, it is difficult for clinicians to explain ML models more accurately and intuitively. Therefore, we developed a regression model using the SHAP method. The advantage of the SHAP is its ability to provide a fair, transparent, and comprehensible method for quantifying the specific contributions of each feature to a model’s predictions, thereby enhancing the interpretability and credibility of the model.

In this study, the SHAP values indicated that capsule invasion, nodule multiplicity, and nodule size are important predictors of cervical lymph node metastasis (CLNM), which is consistent with previous findings (4, 9). Consequently, it is advisable to consider the increased risk of clinical lymph node metastasis in patients with multiple PTCs invading the capsule. In addition, clinical features such as male sex, age < 45 years, and a maximum diameter >1.0 cm, have been identified as high-risk factors for lymph node metastasis (10–12). This study also revealed that age and sex were identified as independent risk factors for CLNM. Furthermore, our study calculated the cut-off value for predicting CLNM based on thyroid nodule size. Notably, we found it to be 0.75 cm, which differs from the previously considered 1 cm threshold. This result indicates that the occurrence of CLNM should be carefully considered in patients who are male, <45 years, and have a nodule diameter greater than 0.75 cm, to prevent inadequate treatment. The irregular shape of the nodule is a sign of malignancy due to extensive fibrosis of the interstitium of the thyroid cancer nodule, causing internal collagen fibrosis of the papillary structures to pull each other, leading to irregular forms. Malignant nodules exhibit infiltrative growth, often resulting in unclear boundaries. Therefore, when a nodule is close to or breaks through the envelope, despite not having the irregular shape, lymph node dissection should be considered in clinical practice.

Studies have shown that serum markers can predict the biological behavior of lymph node metastases and PTC (13). Hu et al. (14) confirmed that FT4 and TSH levels are positively associated with PTC. Serological studies on autoimmune antibodies have revealed that the incidence of HT has increased significantly in patients with thyroid cancer in recent years (15, 16); however, different researchers have reached conflicting conclusions regarding the relationship between HT background and the occurrence of lymph node metastases (17–19). Our study revealed that the background of HT is not a risk factor for lymph node metastasis in patients with thyroid cancer, which is inconsistent with the findings of Li, suggesting that the correlation between the high expression status of TgAb and TPOAb and the occurrence of lymph node metastasis in patients with thyroid cancer still needs to be further explored. By contrast, an autoimmune response to HT can lead to increased TSH levels, which may promote the growth and invasion of PTC (20, 21). No correlation was found between lymph node metastasis and TSH levels in this study, which is the source of controversy among doctors of different clinical specialties regarding the treatment of intermediate-risk PTC with TSH suppression and HT. Accordingly, additional high-quality studies are warranted to inform and optimize the clinical management of thyroid cancer. Although some scholars have suggested that the BRAF^V600E^ mutation phenotype is a hazard for lymph node metastasis (22), the results of common studies do not support this finding, which is the current mainstream research conclusion (23). Our study revealed statistically significant differences in BRAF^V600E^ mutation among various lymph node metastasis subgroups; however, this characteristic did not emerge as an independent risk factor for CLNM. However, BRAF^V600E^ mutation detection has important adjunctive diagnostic value in distinguishing benign and malignant thyroid nodules (24).

Our study had several limitations. First, this was a retrospective study, and selection bias was unavoidable. Second, the number of patients with PTC included in this study was relatively small. Thus, although high consistency in the reproducibility analysis of the training and test groups was achieved, there may be some unavoidable errors due to the uncertainty of data segmentation. In the future, we will include more cases for further verification. The interpretation of ultrasound image features largely depends on the operator’s scanning habits and the diagnostician’s experience. Therefore, the presence of subjective factors may have affected the final data.

## 5 Conclusion

In summary, we constructed a predictive model based on the ML model and found that the logistic model performed better in this study. Our model exhibits robust predictive performance across various subgroups of lymph node metastasis, highlighting its potential utility in clinical settings. In addition, we performed personalized risk assessment for cervical lymph node metastasis in patients with PTC. This effective computer-assisted method can further help clinicians and patients to identify the occurrence of lymph node metastasis and provides a foundation for developing strategies to guide surgical decisions and enhance patient prognosis prior to clinical surgery.

## Data Availability

The raw data supporting the conclusions of this article will be made available by the authors, without undue reservation.

## Supplementary material

Supplementary Figure 1 The diagnostic efficacy of optimal model across different subgroups. **(A)** The ROC curve of the optimal model in predicting the subgroup with CLNM. **(B)** The ROC curve of the optimal model in predicting the subgroup with LCLNM.

Supplementary Figure 2 Model performance on the external validation set. **(A)** ROC curve in the training set, **(B)** ROC curve in the external validation set, and **(C)** calibration curve.
