# Supplementary figures and images for: Construction and validation of a predictive model for lymph node metastasis in patients with papillary thyroid carcinoma
Source: Front Endocrinol (Lausanne). 2025 Jun 9;16:1551108. doi: 10.3389/fendo.2025.1551108 (PMC12183031; doi:10.3389/fendo.2025.1551108)

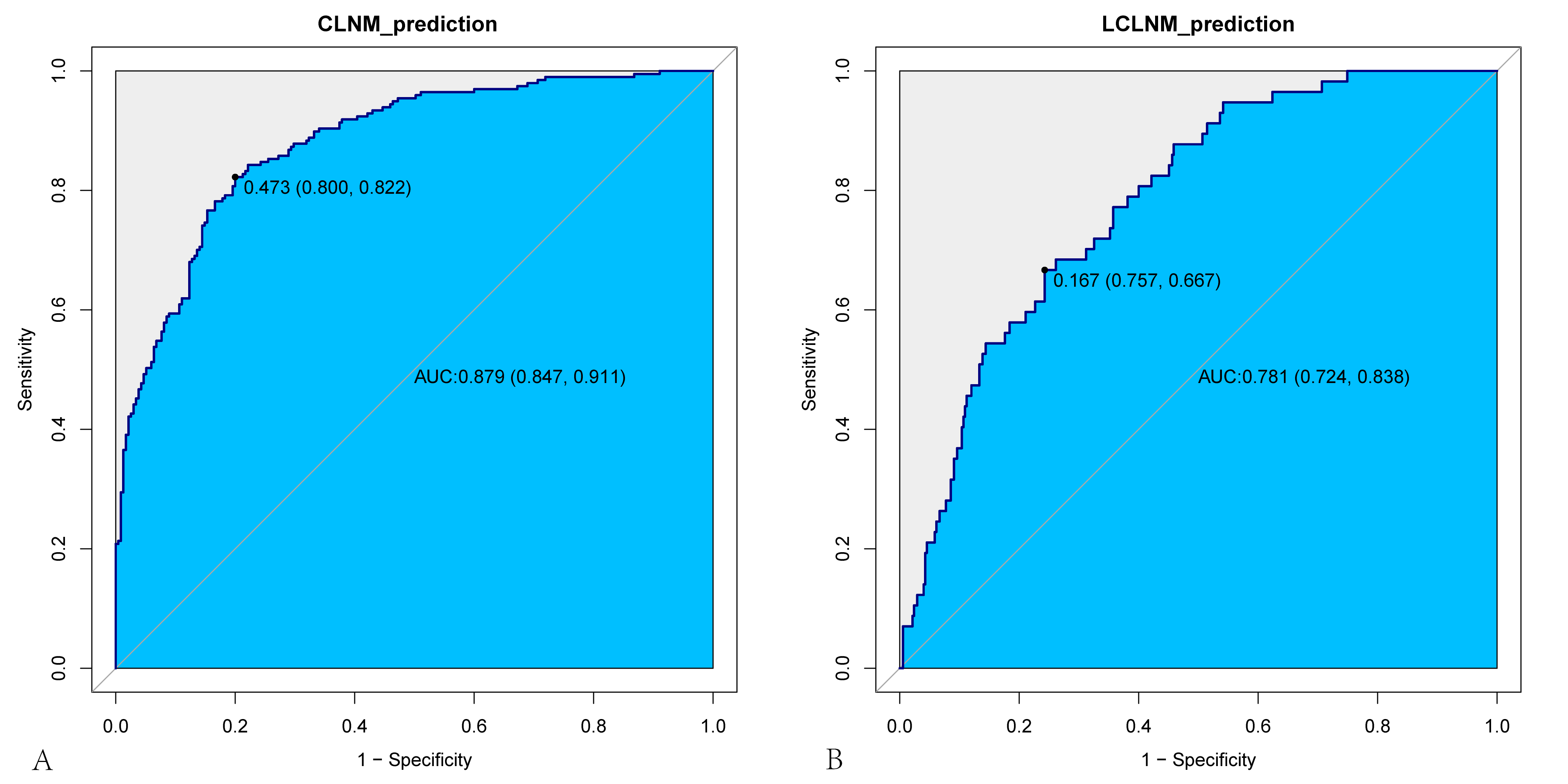

Supplement: Supplementary Figure 1 — The diagnostic efficacy of optimal model across different subgroups. (A) The ROC curve of the optimal model in predicting the subgroup with CLNM. (B) The ROC curve of the optimal model in predicting the subgroup with LCLNM. [file Image1.tif]

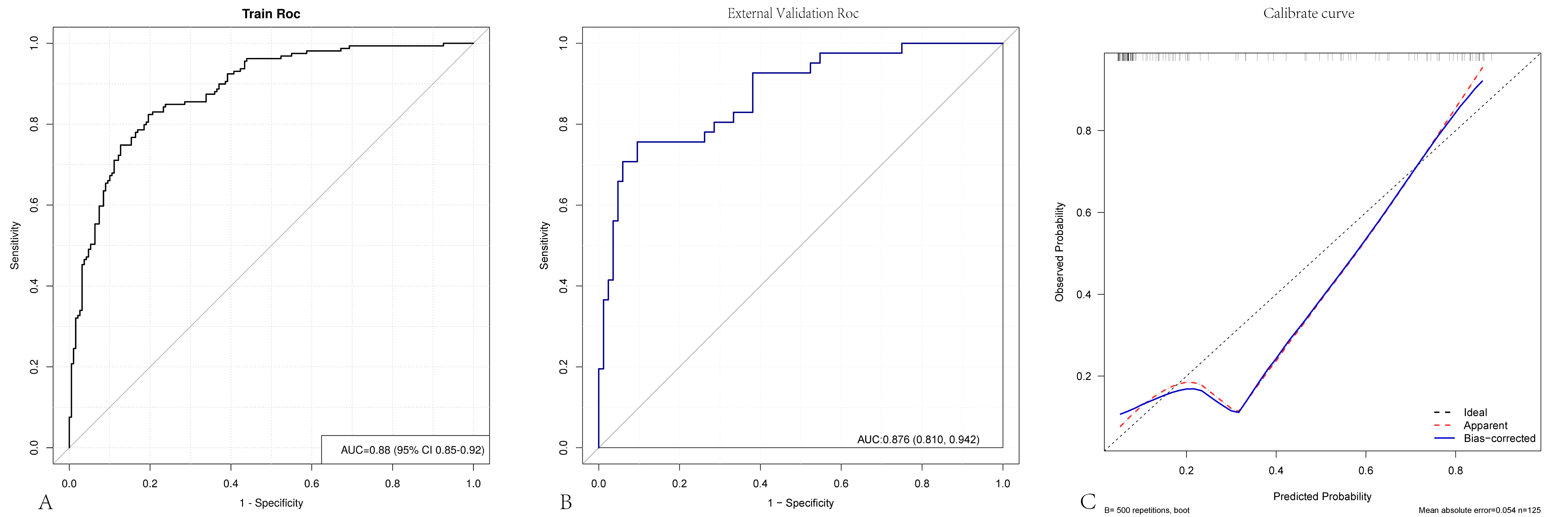

Supplement: Supplementary Figure 2 — Model performance on the external validation set. (A) ROC curve in the training set, (B) ROC curve in the external validation set, and (C) calibration curve. [file Image2.tif]
