# Supplementary material for: Construction and validation of a predictive model for lymph node metastasis in patients with papillary thyroid carcinoma
Source: Front Endocrinol (Lausanne). 2025 Jun 9;16:1551108. doi: 10.3389/fendo.2025.1551108 (PMC12183031; doi:10.3389/fendo.2025.1551108)
Supplement: Supplementary file 3 [file SupplementaryFile1.doc]

Supplementary Table 1 Baseline of PTC with Different Lymph Node Statuses

| Variables | Total | Negative | CLNM | LCLNM | p |
| --- | --- | --- | --- | --- | --- |
|  | (n = 432) | (n = 235) | (n = 140) | (n = 57) |  |
| Age (year) | 45.6 ± 11.7 | 47.9 ± 11.2a | 42.9 ± 11.3b | 42.4 ± 12.5b | < 0.001 |
| Gender |  |  |  |  | 0.014 |
| Male | 94 (21.8) | 39 (16.6)a | 41 (29.3)b | 14 (24.6)ab |  |
| Female | 338 (78.2) | 196 (83.4)a | 99 (70.7)b | 43 (75.4)ab |  |
| BRAF^V600E^ mutation |  |  |  |  | < 0.001 |
| No | 39 ( 9.0) | 39 (16.6)a | 0 (0)b | 0 (0)b |  |
| Yes | 393 (91.0) | 196 (83.4)a | 140 (100)b | 57 (100)b |  |
| FT3 |  |  |  |  | 0.129 |
| Normal | 408 (94.4) | 226 (96.2) | 131 (93.6) | 51 (89.5) |  |
| Abnormal | 24 ( 5.6) | 9 (3.8) | 9 (6.4) | 6 (10.5) |  |
| FT4 |  |  |  |  | 0.330 |
| Normal | 403 (93.3) | 223 (94.9) | 128 (91.4) | 52 (91.2) |  |
| Abnormal | 29 ( 6.7) | 12 (5.1) | 12 (8.6) | 5 (8.8) |  |
| TSH |  |  |  |  | 0.028 |
| Normal | 333 (77.1) | 176 (74.9)ab | 118 (84.3)a | 39 (68.4)b |  |
| Abnormal | 99 (22.9) | 59 (25.1)ab | 22 (15.7)a | 18 (31.6)b |  |
| Tg |  |  |  |  | 0.028 |
| Normal | 396 (91.7) | 219 (93.2)a | 130 (92.9)ab | 47 (82.5)b |  |
| Mild abnormality | 19 ( 4.4) | 11 (4.7)a | 5 (3.6)a | 3 (5.3)a |  |
| Significant abnormality | 17 ( 3.9) | 5 (2.1)a | 5 (3.6)ab | 7 (12.3)b |  |
| HT |  |  |  |  | 0.616 |
| No | 380 (88.0) | 209 (88.9) | 123 (87.9) | 48 (84.2) |  |
| Yes | 52 (12.0) | 26 (11.1) | 17 (12.1) | 9 (15.8) |  |
| Margin |  |  |  |  | < 0.001 |
| Clear | 50 (11.6) | 40 (17)a | 4 (2.9)b | 6 (10.5)ab |  |
| Unclear | 382 (88.4) | 195 (83)a | 136 (97.1)b | 51 (89.5)ab |  |
| Relationship to capsule |  |  |  |  | < 0.001 |
| Away from | 137 (31.7) | 123 (52.3)a | 11 (7.9)b | 3 (5.3)b |  |
| Adjacent | 182 (42.1) | 87 (37)a | 71 (50.7)b | 24 (42.1)ab |  |
| Invasion | 113 (26.2) | 25 (10.6)a | 58 (41.4)b | 30 (52.6)b |  |
| Number |  |  |  |  | < 0.001 |
| Solitary | 244 (56.5) | 156 (66.4)a | 63 (45)b | 25 (43.9)b |  |
| Multiple | 188 (43.5) | 79 (33.6)a | 77 (55)b | 32 (56.1)b |  |
| Composition |  |  |  |  | 0.017 |
| Solid | 394 (91.2) | 222 (94.5)a | 125 (89.3)ab | 47 (82.5)b |  |
| Mixed solid and cystic | 38(8.8) | 13(5.5)a | 15(10.7)ab | 10(17.5)b |  |
| Echo |  |  |  |  | 0.002 |
| Hypo | 388 (89.8) | 222 (94.5)a | 119 (85)b | 47 (82.5)b |  |
| Isoechoic or hyper | 44 (10.2) | 13 (5.5)a | 21 (15)b | 10 (17.5)b |  |
| Coarse calcification |  |  |  |  | 0.097 |
| No | 301 (69.7) | 174 (74) | 90 (64.3) | 37 (64.9) |  |
| Yes | 131 (30.3) | 61 (26) | 50 (35.7) | 20 (35.1) |  |
| Aspect ratio |  |  |  |  | 0.005 |
| <1 | 100 (23.1) | 41 (17.4)a | 39 (27.9)ab | 20 (35.1)b |  |
| ≥1 | 332 (76.9) | 194 (82.6)a | 101 (72.1)ab | 37 (64.9)b |  |
| Shape |  |  |  |  | < 0.001 |
| Regular | 126 (29.2) | 94 (40)a | 20 (14.3)b | 12 (21.1)b |  |
| Irregular | 306 (70.8) | 141 (60)a | 120 (85.7)b | 45 (78.9)b |  |
| Microcalcification |  |  |  |  | < 0.001 |
| No | 77 (17.8) | 57 (24.3)a | 16 (11.4)b | 4 (7)b |  |
| Yes | 355 (82.2) | 178 (75.7)a | 124 (88.6)b | 53 (93)b |  |
| Size (cm) | 0.8 (0.6, 1.3) | 0.7 (0.6, 1.0)a | 1.0 (0.7, 1.5)b | 1.3 (0.9, 1.9)c | < 0.001 |
| Position (upper-lower distribution) |  |  |  |  | 0.004 |
| Isthmus | 18 ( 4.2) | 8 (3.4)a | 10 (7.1)a | 0 (0)a |  |
| Upper | 69 (16.0) | 39 (16.6)a | 17 (12.1)a | 13 (22.8)a |  |
| Middle | 183 (42.4) | 100 (42.6)a | 55 (39.3)a | 28 (49.1)a |  |
| Lower | 82 (19.0) | 53 (22.6)a | 26 (18.6)ab | 3 (5.3)b |  |
| Diffuse occurrence | 80 (18.5) | 35 (14.9)a | 32 (22.9)a | 13 (22.8)a |  |
| Location (left-right distribution) |  |  |  |  | 0.082 |
| Isthmus | 18 ( 4.2) | 8 (3.4) | 10 (7.1) | 0 (0) |  |
| Left | 141 (32.6) | 79 (33.6) | 42 (30) | 20 (35.1) |  |
| Right | 193 (44.7) | 113 (48.1) | 56 (40) | 24 (42.1) |  |
| Diffuse occurrence | 80 (18.5) | 35 (14.9) | 32 (22.9) | 13 (22.8) |  |

Note: different letters in the same row indicate significant differences.

Supplementary Table 2 Diagnostic performance of the predictive model across different subgroups

| Subgroups | AUC | 95%CI | Specificity | Sensitivity | NPV | PPV | Jordon's index |
| --- | --- | --- | --- | --- | --- | --- | --- |
| LCLNM | 0.7813 | 0.7244 – 0.8383 | 0.7573 | 0.6667 | 0.9373 | 0.2946 | 0.424 |
| CLNM | 0.8790 | 0.8472 – 0.9108 | 0.8000 | 0.8223 | 0.843 | 0.7751 | 0.6223 |
